# Anthelmintic efficacy evaluation and mechanism of *N*-methylbenzo[d]oxazol-2-amine

**DOI:** 10.1038/s41598-023-50305-y

**Published:** 2023-12-21

**Authors:** Pattaneeya Prangthip, Jumreang Tummatorn, Poom Adisakwattana, Naphatsamon Uthailak, Usa Boonyuen, Phornpimon Tipthara, Joel Tarning, Pavitra Laohapaisan, Charnsak Thongsornkleeb, Somsak Ruchirawat, Onrapak Reamtong

**Affiliations:** 1 Department of Tropical Nutrition and Food Science, Faculty of Tropical Medicine, Mahidol University, Bangkok, 10400 Thailand; 2 Program on Chemical Sciences, Chulabhorn Graduate Institute, Center of Excellence on Environmental Health and Toxicology (EHT), OPS, MHESI, 54 Kamphaeng Phet 6, Laksi, Bangkok, 10210 Thailand; 3 Laboratory of Medicinal Chemistry, Chulabhorn Research Institute, 54 Kamphaeng Phet 6, Laksi, Bangkok, 10210 Thailand; 4 Department of Helminthology, Faculty of Tropical Medicine, Mahidol University, Bangkok, 10400 Thailand; 5 Department of Social and Environmental Medicine, Faculty of Tropical Medicine, Mahidol University, Bangkok, 10400 Thailand; 6 Department of Molecular Tropical Medicine and Genetics, Faculty of Tropical Medicine, Mahidol University, Bangkok, 10400 Thailand; 7 Mahidol Oxford Tropical Medicine Research Unit, Faculty of Tropical Medicine, Mahidol University, Bangkok, 10400 Thailand; 8 Centre for Tropical Medicine and Global Health, Nuffield Department of Clinical Medicine, University of Oxford, Oxford, UK

**Keywords:** Molecular biology, Diseases

## Abstract

Parasitic roundworms cause significant sickness and mortality in animals and humans. In livestock, these nematodes have severe economic impact and result in losses in food production on a global scale. None of the currently available drugs ideally suit all treatment circumstances, and the development of drug-resistant nematode strains has become a challenge to control the infection. There is an urgent need to develop novel anthelmintic compounds. According to our previous report, *N*-methylbenzo[d]oxazol-2-amine (**1**) showed anthelmintic activity and lowest cytotoxicity. In this study, in vivo anthelmintic properties were evaluated using *Trichinella spiralis* infected mice. Toxicity was evaluated using the rats and mode of action using molecular docking and metabolomics approaches. The in vivo results demonstrate that a dose of 250 mg/kg reduced the *T. spiralis* abundance in the digestive tract by 49%. The 250 mg/kg Albendazole was served as control. The relatively low acute toxicity was categorized into chemical category 5, with an LD_50_ greater than 2000 mg/kg body. Molecular docking analysis showed the *T. spiralis* tubulin beta chain and glutamate-gated channels might not be the main targets of compound **1**. Metabolomics analysis was used to explain the effects of compound **1** on the *T. spiralis* adult worm. The results demonstrated that compound 1 significantly up-regulated the metabolism of purine, pyrimidine and down-regulated sphingolipid metabolism. In conclusion, compound **1** could be a potential molecule for anthelmintic development. The bioavailability, pharmacokinetics, and absorption of this compound should be studied further to provide information for its future efficacy improvement.

## Introduction

Almost 300 nematode species cause zoonotic diseases in humans^1^. Some parasitic nematodes enter the human body through open wounds or penetrate the skin, while others infect people when they are consumed via food products contaminated with embryonated eggs or parasitic nematode larvae. Around 24% of the world's population is infected with parasitic nematodes. *Ascaris lumbricoides*, *Ancylostoma duodenale*, *Gnathostoma spinigerum*, *Halicephalobus gingivalis*, and *Trichinella spiralis* are considered harmful parasites. Not only human, nearly every organ in an animal's body also can harbor nematode parasites. The digestive, circulatory, and respiratory systems are the most frequently infected organs. Eelworm, lungworm, pinworm, threadworm, and hookworm are common found in animals^2^. In order to effectively control the parasitic nematodes, "One Health" approach should be concerned. Since there are no vaccines for parasitic nematodes, the most promising long-term control strategy currently relies on chemotherapeutic medicines to kill parasitic nematodes and limit the spread of infections.

Nematicides are separated into several classes based on similarities in their chemical structures and modes of action. Piperazine, initially used as an anthelmintic in the 1950s, has a GABA-mimetic effect and paralyzes the nematode body muscle^3^. The first benzimidazole class compound discovered was thiabendazole in 1961 and, subsequently, different benzimidazole structures have been introduced as broad spectrum anthelmintics. It is clear that the anthelmintic efficacy of benzimidazoles is through their selective interaction with *β*-tubulin^4^. Levamisole, pyrantel, and morantel are nicotinic receptor agonists that cause the excitation of nicotinic acetylcholine receptors on muscles, resulting in spastic muscular paralysis^5^. Paraherquamide A and marcfortine A are members of the oxindole alkaloid family originally isolated from *Penicillium paraherquei* and *Penicillium roqueforti*, respectively. They both induce paralysis in parasitic nematodes and act as typical competitive antagonists of acetylcholine-stimulated muscle contractions^6^. Avermectins are a macrocyclic lactone endectocides that are produced naturally or semi-synthetically^7^. They are made from the fermentation products of *Streptomyces avermitilis*, a soil-dwelling actinomycete that is able to efficiently control a wide range of endo- and ectoparasites^8^. This drug and its analogs demonstrate inhibitory activity on glutamate-gated chloride channels (GluCl), leading to the inhibition of pharyngeal pumping, motility, and egg or microfilaria release and a loss of host immunosuppression^9^. Milbemycins are products of Streptomyces species' fermentation. They action by a similar mechanism, but their half-life is longer than that of avermectins. Invertebrate neurons and myocytes' glutamate-sensitive chloride channels are opened, which causes these cells to become hyperpolarized and impairs signal transmission^10^. Derivatives of aminoacetonitrile are effective helminthicides. They function as nematode-specific nematode ACh agonists, resulting in a spastic paralysis and quick ejection from the host^11^. The cyclodepsipeptide molecule emodepside is a semi-synthetic derivative of PF1022A, a fermentation product obtained from a fungus, *Mycelia Sterilia*. The emodepside causes paralysis in nematodes by stimulating excessive neurotransmitter release at neuromuscular sites^12^. Nitazoxanide, a pyruvate ferredoxin oxidoreductase inhibitor, acts against a broad spectrum of protozoa and helminths that occur in the intestinal tract, and anaerobic electron transport enzymes are its potential targets^13^.

**Figure 1:**
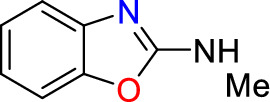
Structure of *N*-methylbenzo[d]oxazol-2-amine (**1**).

Because of the extraordinary effectiveness of modern anthelmintics, with more than 95% parasite reduction, as well as their generally good safety margin, broad-spectrum nature, and reasonable pricing, the chemical control of parasites in animals has been very successful over the past 50 years. Unfortunately, the prolonged use of these therapies and inappropriate drug doses have resulted in the growth of drug resistance among parasitic nematodes, primarily the gastrointestinal nematodes of cattle, sheep, goats, and horses^14^. Currently, instances of nematode resistance to all available anthelmintic drugs, including piperazine, benzimidazoles, levamisole, paraherquamide, ivermectin (IVM), emodepside, nitazoxanide, milbemycins and aminoacetonitrile derivatives have been documented^15,16^. In a study in Argentina, resistance was determined in vivo by a fecal egg count reduction test. The resistances for ivermectin, albendazole and levamisole were 60%, 32% and 28%, respectively. A high level of anthelmintic resistance was observed in this study^17^. In New Zealand, a cross-sectional prevalence study was performed using a standardized fecal nematode egg count reduction test. Resistance to ivermectin, ivermectin-levamisole, ivermectin-albendazole and ivermectin-levamisole-albendazole were found on 36, 10, 13 and 8% farms, respectively. The prevalence of resistance to ivermectin was showed high prevalent on these farms^18^. Moreover, climate change has already affected parasite levels and species composition, and large-scale outbreaks have become more frequent, endangering both animal welfare, food security, and public health^19^. Therefore, there is an urgent need for further anthelmintic drug development. According to our previous study, *N*-methylbenzo[d]oxazol-2-amine (**1**) has good anthelmintic properties. The chemical structure of this compound is different from that of known anthelmintic drugs (Fig. 1). An in vitro study indicated they have comparable potency to albendazole (ABZ) in a free-living nematode *Caenorhabditis elegans* strain N2 and parasitic nematodes (*T. spiralis* muscle stage and third-stage *Gnathostoma spinigerum* larvae)^20^. The 50% effective concentration (EC_50_) of compound **1** on *C. elegans* and *T. spiralis* were 5.77 and 3.8 µM, respectively. In addition, compound **1** demonstrated low cytotoxicity at 50% cytotoxic concentration of 1282 mM to human embryonic kidney 293 cell (HEK293)^20^. In fact, compound **1** showed about 10 times lower cytotoxicity towards human embryonic kidney cells than ABZ.

Since the complete life cycle of *T. spiralis* could be simply maintained in the laboratory, it does not require additional intermediate host and alternative non-laboratory animals. In addition, it could provide two important parasite stages including muscle and intestinal stages which could be used for researching*.* Number of worms in each batch are also sufficient for drug screening experiment. Therefore, *T. spiralis* was selected as a parasitic nematode model in this study.

The in vivo anthelmintic efficiency, toxicity in animal models, and potential mechanism of action of compound **1** were evaluated. The in vivo infection of the adult parasitic worm *T. spiralis* in an animal model was used to test the effectiveness and toxicity of anthelmintics. Our findings provide the necessary information to evaluate the potential of compound **1** as a treatment for helminth infections.

## Material and methods

### Compound

All *N*-Methylbenzo[d]oxazol-2-amine in this study was evaluated its purity by an Agilent High Performance Liquid Chromatography (HPLC). HPLC Column Hichrom, C18, 150 Å, 5 µm, 4.6 × 250 mm was used for separation with 0.5ml/min flowrate. Mobile phase A and B were water and isopropanol, respectively. The gradient was started from 30%B to 95%B in 77 min. A diode array was the detector at 254nm absorbance. The HPLC trace of compound **1** was > 95% pure (Fig. S1).

### Anthelmintic activity on *T. spiralis* adult worm

The experimental procedures were approved by the Faculty of Tropical Medicine Animal Care and Use Committee, Mahidol University (Approval number: FTM-ACUC 033/2020). All animal experiments were conducted in accordance with ARRIVE guidelines. All methods were performed in accordance with relevant guidelines and regulations. The laboratory strain of *T. spiralis* used in this study was maintained in the Animal Care Unit, Faculty of Tropical Medicine, Mahidol University. For the in vitro anthelmintic assay, 8-week-old female ICR mice (three mice) were fed 250 larvae by oral gavage. After 7 days of infection, the mice were euthanized by compressed carbon dioxide gas. Adult worms were collected from the digestive tracts by flushing with normal saline. The worms were cultured in RPMI 1640 media, with L-glutamine (SH30027.01) purchased from Hyclone (Logan, UT, USA) for anthelmintic testing. Compound **1** concentrations in RPMI were generated in triplicate, from 10 ng/mL to 100 μg/mL, and used to calculate the half maximum effective concentration (EC_50_) (final concentration in wells with 0.5% DMSO). Positive was ABZ (CAS No. 54965-21-8, purity ≥ 98.0%) purchased from Sigma-Aldrich (MO, USA) with the same concentration range with compound **1**. While, 0.5% DMSO (Sigma-Aldrich, MO, USA) was negative control in this experiment. Each well received 20 worms, which were then cultured at 37 °C for 24 h. Worm motility was assessed under an inverted microscope. Data were shown as the mean ± S.E.M. two-way ANOVA was used (with significance level set at P < 0.05). All statistics were carried out using GraphPad Prism software. GraphPad Prism 9 software was used to calculate the EC_50_. The molecular weights of compounds were used to calculate EC_50_ to µM unit.

For the in vivo anthelmintic assay, 8-week-old female ICR mice (six mice/group) weighing 30 ± 3 g from Nomura Siam International Co., Ltd., (Thailand, Bangkok) were fed 250 larvae by oral gavage after 7 days acclimatization. After 24 h of infection, single oral doses of 250, 500, and 1000 mg/kg compound **1** were fed to the infected mice by oral gavaging of the suspension in the tween 80 (15% v/v) (Sigma-Aldrich, MO, USA). Water and 250 mg/kg ABZ were administered to the negative and positive controls, respectively. After 7 days of infection, adult worms were recovered from mouse digestive tracts as described above. The number of worms was evaluated under an inverted microscope.

### Acute oral toxicity

The experimental procedures were approved by the Faculty of Tropical Medicine Animal Care and Use Committee, Mahidol University (Approval number: FTM-ACUC 006/2020). All animal experiments were conducted in accordance with ARRIVE guidelines. All methods were performed in accordance with relevant guidelines and regulations. Sprague–Dawley (SD) rats, 5 weeks old, weighing 109 ± 5.62 g, from Nomura Siam International Co., Ltd. (Thailand, Bangkok) were used for toxicity testing according to Organization for Economic Co-operation and Development (OECD) Guidelines for the Testing of Chemicals 425. After 7 days acclimatized at the room temperature of 22 ± 2 °C, relative humidity of 55 ± 5%, light and dark cycle of 12:12 h, each rat was fasted for approximately 16 h before compounds **1** gavage. The compounds were dissolved in a 15% v/v solution of Tween 80 and orally administered to rats at doses of 0, 175, 550, and 2000 mg/kg body weight, in accordance with the OECD 425 guidelines, with administration conducted at 10:00 a.m. During testing, unlimited feeding a standard diet and water are provided until the end of the 14-day period. Then, each rat was fasted for approximately 16 h and euthanized with carbon dioxide. Serum samples were then collected for glucose, lipid and general health indicators using an automated blood sample testing instrument (Cobas analyzer, Roche Diagnostics, Switzerland). Organs including the spleen, kidney and liver were collected and weighed. longitudinally Longitudinal sections were immersed overnight in 10% buffer formalin for biopsy and stained with hematoxylin and eosin for microscopic study. Weight and blood biochemical test data are presented as means ± standard deviations (SD). Data were analyzed using one-way analysis of variance (ANOVA) following with Tukey’s range tests (SPSS Statistics software Version 18, NY, USA). Statistical significance was accepted when P < 0.05.

### Docking compound **1** with ABZ and IVM target proteins

Three-dimensional (3D) models of tubulin beta chain and glutamate-gated chloride channel (GluCl) from *Trichinella spiralis* were constructed by SWISS-MODEL^21^. The tubulin beta of decorated ciliary doublet microtubule (PDB ID: 6U42) and the *C. elegans* GluCl (PDB ID: 4TNW) were used as templates for tubulin beta chain and GluCl, respectively. *T. spiralis* tubulin beta chain and GluCl shared 87.10% and 60.06% sequence identity with the templates, respectively. Subsequently, the obtained 3D models were validated using SAVESv6.0 (https://servicesn.mbi.ucla.edu/SAVES/). Albendazole and *N*-methylbenzo[d]oxazol-2-amine and ivermectin and *N*-methylbenzo[d]oxazol-2-amine were docked to the *T. spiralis* tubulin beta chain and GluCl, respectively, by Autodock Vina^22^ using CB-Dock server^23^.

### Metabolomics analysis

For metabolite extraction, 0.5% DMSO and EC_50_ compound **1** treated *T. spiralis* adult worms were homogenized in 500 μL methanol. The tubes were liquid nitrogen-snap frozen, thawed, then centrifuged at 800*g* for one minute at 4 °C. The pellet was extracted once more using the same procedure. The supernatant was collected and put in a fresh tube. The second extraction's supernatant was added into the first extraction's supernatant. The pellet was redissolved in 250 μL of deionized water, the pellet was frozen in liquid nitrogen and thawed. Centrifugation at 15,000*g* for 1 min at 4 °C was used to collect the supernatant, which was then combined into the previous extraction. To get rid of the last bits of debris, the pooled supernatants were centrifuged at 15,000*g* for one minute at 4 °C. The clear supernatant was collected and dried in a speed vacuum (Tomy Digital Biology, Tokyo, Japan). The metabolite pellet was reconstituted in 200 μL of mobile phase A:B at a ratio of 50:50 (vol/vol) and subjected to the ultra-high performance liquid chromatography (UHPLC; Agilent 1260 Quaternary pump, Agilent 1260 High Performance Autosampler, and Agilent 1290 Thermostatted Column Compartment SL, Agilent Technologies) coupled to a quadrupole time-of-flight mass spectrometer (Q-TOF-MS) (TripleTOF 5600+, SCIEX, US). The mobile phase A and B was water containing 0.1% formic acid and acetonitrile containing 0.1% formic acid, respectively. A C18 reversed phase column (ACQUITY UPLC HSST3, 2.1 × 100 mm, 1.8 μM, Waters) protected by a pre-column (ACQUITY UPLC HSST3, 2.1 × 5 mm, 1.8 μM, Waters) was used for separation with a flow rate of 0.3 mL/min at 40 °C. The gradient was started at 5% mobile phase B for 2.0 min (0.0–2.0 min), 5–60% B for 0.5 min (2.0–2.5 min), 60–80% B for 1.5 min (2.5–4.0 min), 80–100% B for 8.0 min (4.0–12.0 min), 100% B for 5 min (12.0–17.0 min), 100–5% B for 0.1 min (17.0–17.1 min), and 5% B for 2.9 min (17.1–20.0 min). The UHPLC-Q-TOF-MS system, mass ion chromatogram, and mass spectra were acquired by Analyst Software version 1.7 (SCIEX). The Q-TOF-MS was operated in positive (+ESI) and negative (−ESI) electrospray ionization modes. Equal aliquots of each metabolite sample were pooled to form the quality control (QC) samples. The QC samples were injected before, during, and after sample analysis to assess the system performance. Raw mass spectra files from the UHPLC-Q-TOF-MS (.wiff and .wiff.scan files) were processed using the XCMS online software version 3.7.1 (The Scripps Research Institute, CA, USA). The comparison between control and treated groups were performed using “Pairwise” mode with “UPLC/Triple TOF pos” protocol. Metabolomic data from XCMS were then analyzed using MetaboAnalyst online software version 5.0 (https://www.metaboanalyst.ca/)^24^ in both “Statistical Analysis (one factor)” and “Pathway Analysis” modules. For statistical analysis module, metabolites with their concentrations were filtered by “Interquantile range (IQR)” and normalized using quantile normalization, cube root data transformation, and data range scaling. Data visualization was performed using Partial Least Squares-Discriminant Analysis (PLS-DA), and Volcano plot. PLS-DA were demonstrated with 95% confidence regions. Log2 of fold change and -log of p-value were used to generate the Volcano plot. Differential metabolites were identified with the specific cutoff (> 1.5-fold change, p-value < 0.01). Pathway analysis of differential metabolites was performed in MetaboAnalyst and the STITCH database version 5.0 (http://stitch.embl.de/)^25^ with the p-value less than 0.01 as a statistical significance.

### Compliance with ethics guidelines

The animal study were approved and performed in complete compliance with the ethical approval granted by the Faculty of Tropical Medicine Animal Care and Use Committee, Mahidol University (Approval number: FTM-ACUC 006/2020 and FTM-ACUC 033/2020).

## Results and discussion

### Anthelmintic activity on *T. spiralis* adults

In this study, *T. spiralis* adult worms served as a model intestinal parasitic nematode. *T. spiralis* is a parasitic worm with a complex life cycle and sophisticated gene regulation during development, and employing *T. spiralis* as a model organism may help with the development of drugs^26^. The EC_50_ of compound **1** was examined by in vitro assay 1, 2, 3, and 24 h after exposure, and ABZ was applied as positive-control drug. The in vitro anthelmintic activity results are presented in Table 1. According to the results, no *T. spiralis* adult worms were dead 1 and 2 h after compound **1** or ABZ exposure. Worm motility was evaluated under an inverted microscope. Living nematodes maintain a sinusoidal shape and show locomotion, whereas alive paralysis is characterized by a rod-shaped body posture with integrity and structure of internal organs. The dead nematodes appear as straight, rigid rods and complete disintegration of internal organs. Data were shown as the mean ± S.E.M. two-way ANOVA was used (with significance level set at P < 0.05). Interestingly, the EC_50_ of compound **1** was 208.6 µM, which was statistically lower than that of ABZ (275.05 µM), after 3 h of exposure. In addition, the EC_50_ of compound **1** was 1.55 µM, which was lower than that of ABZ (3.09 µM), after 24 h of exposure. These in vitro assay findings demonstrated that compound **1** has an approximately twofold greater activity than ABZ at 24 h. In this study, the latest time exposure was 24 h followed the protocol of Ruo Yu Peng et al.^27^. The longer exposure time might provide better results. In comparison to previous study, at 24 h after treatment, the EC_50_ of compound **1** was 3.8 µM, which was approximately threefold less activity than ABZ (1.3 µM). This compound showed better anthelmintic activity in adult than larvae stages. One of limitation of this study was the random selection of adult worm genders. The different worm gender may affect the anthelminthic activity. Therefore, the gender specific experiment should be further performed to evaluate the compound **1** activity.

**Table 1:** EC_50_ of compound 1 on in vitro* T. spiralis* adult worms 1, 2, 3, and 24 h after exposure.

Time (h)	EC_50_	
	ABZ (*µ*M)	Compound **1** (*µ*M)
1	n/a	n/a
2	n/a	n/a
3	275.05 ± 5.78	208.6 ± 7.14*
24	3.09 ± 0.39	1.55 ± 0.17*

Three biological replicates were performed and the average and standard deviation of EC_50_ are presented in the table.

n/a: *T. spiralis* adult worms were not dead at 1 and 2 h after compound 1 or ABZ exposure.

Data were shown as the mean ± S.E.M. two-way ANOVA was used (* significance level at P < 0.05).

As compound **1** demonstrated promising activity in vitro, the effectiveness of compound **1** as an anthelmintic was evaluated in animal models. Six infected mice per group were used in this study. The infected mice were given single oral doses of 250, 500, or 1000 mg/kg of compound **1**. The in vivo efficacy of compound **1** is shown in Table 2. Since an appropriate dose of ABZ to use against *T. spiralis* in muscle is 250 mg/kg^28,29^ mice treated with a single oral dose of 250 mg/kg ABZ were included as positive controls. The oral doses of compound **1** was started from the same concentration of ABZ and increased two-fold more concentration to improve efficiency. Untreated infected mice were used as the negative controls.

**Table 2:** Percentage worm reduction after feeding mice with single oral dose of 250, 500, or 1000 mg/kg compound **1.**

Group	% reduction
Infected mice (control)	0.00
250 mg/kg ABZ-treated mice	100.00 ± 0.00*
250 mg/kg compound **1**-treated mice	49.17 ± 4.71*
500 mg/kg compound **1**-treated mice	64.54 ± 6.85*
1000 mg/kg compound **1**-treated mice	76.50 ± 7.22*

Six biological replicates were performed and the average and standard deviation of EC_50_ are presented in the table.

Data were shown as the mean ± S.E.M. two-way ANOVA was used (* significance level at P < 0.05).

The results showed that compound **1** reduced worm abundance in the digestive tracts of the infected mice by 49.17%, 64.54%, and 76.50% when we administrated compound **1** at 250, 500, and 1000 mg/kg, respectively. The anthelmintic efficacy improved when we increased the dose of compound **1** and was statistically different from control (infected mice). However, ABZ cleared all adult worms from the digestive tract of mice at 250 mg/kg. The in vivo anthelmintic efficacy of compound **1** was, therefore, not comparable to that of ABZ. One limitation of this experiment was that compound 1 or ABZ was fed to the mice after 24 h of infection and recorded the adult worm viability after 7 days of infection. The worms might not totally change to adult stage on the day that we fed compounds. Therefore, the result demonstrated the compound **1** anthelmintic activity of mix stages between pre-adult and adult of *T. spiralis* at 24 h after infection.

### Toxicity in animal models

In our study, the toxicity assay in the rat model followed the OECD guidelines for the testing of chemicals^30^. For acute oral toxicity, the up-and-down procedure (Test no. 425 from the OECD guidelines) was applied. The method allows for the estimation of a 50% lethal dose (LD_50_) with a confidence interval. A substance can be categorized for acute toxicity in accordance with the Globally Harmonized System of classification and labeling of chemicals. According to the starting dose recommended in the guidelines, 175 mg/kg body weight (BW) of compound **1** was the starting dose used for the rats (n = 5). This dose did not cause changes in the rats’ food and water consumption or excretion over a 14-day experimental period. The dose was progressively increased in accordance with the guidelines. In the first 6 h after applying 550 mg/kg BW, all rats experienced sleepiness and were responsive to their surroundings. In the first 12 h after the concentration was increased to 2000 mg/kg BW, all rats experienced drowsiness, did not consume water or food, and were less responsive to their surroundings. After the first 6 and 12 h and throughout the experimental period, all rats administered doses of 550 and 2000 mg/kg BW, respectively, exhibited normal responses to their surroundings and consumption of food and water. The Korea Institute of Drug Safety & Risk Management monitors drug-safety-related events and collects nationwide reports, and they found that sleepiness, vomiting, dizziness, and nausea were induced by ABZ in the Korean population^31^. In 828 studies, praziquantel was also found to lead to common adverse effects, for example drowsiness, abdominal pain, headache, fatigue, nausea, dizziness, and weakness^32^. Thus the adverse effects of compound **1** are similar to those observed for ABZ and praziquantel.

The BW and organs of rats fed compound **1** concentrations of 175, 550, and 2000 mg/kg BW were not significantly different compared with those given 0 mg/kg BW compound **1**. The rat liver, spleen, and kidney weights were also not significantly different between the groups (Table 3). No deaths were found in any rats in the 14 days after the administration of compound **1** at 0–2000 mg/kg BW. Therefore, we concluded that the acute oral toxicity of compound **1** is represented by an LD_50_ (oral, female rat) greater than 2000 mg/kg body weight. ABZ has been reported to have an LD_50_ of 1320–2400 mg/kg^33^. Therefore, compound **1** showed comparable acute oral toxicity to ABZ.

**Table 3:** Body weight of rats treated with different concentrations of compound 1.

Weight (g)	Compound 1 concentration (mg/kg BW)			
	0	175	550	2000
Initial BW	140.00 ± 9.64	142.30 ± 6.72	141.40 ± 13.68	132.80 ± 7.57
BW at day 7	171.33 ± 4.16	178.20 ± 6.02	173.00 ± 6.40	176.40 ± 6.99
BW at day 14	196.67 ± 14.22	201.20 ± 9.50	208.60 ± 7.92	205.20 ± 8.53
Liver	7.73 ± 0.10	7.96 ± 0.74	7.65 ± 0.64	7.90 ± 0.50
Spleen	0.49 ± 0.12	0.53 ± 0.13	0.51 ± 0.04	0.47 ± 0.05
Right kidney	0.99 ± 0.09	0.95 ± 0.06	0.94 ± 0.06	0.96 ± 0.05
Left kidney	0.93 ± 0.07	0.91 ± 0.04	0.96 ± 0.06	0.95 ± 0.10
Heart	0.76 ± 0.01	0.79 ± 0.11	0.78 ± 0.02	0.75 ± 0.06

Results are reported as mean ± S.E.

Blood chemistry tests provide information on the function of organs, such as the heart, kidneys, and liver^34^. Serum sugar, total cholesterol, triglycerides, high-density lipoprotein, and low-density lipoprotein were not significantly different between the groups. However, rats that received compound **1** concentrations of 175, 550, and 2000 mg/kg body weight tended to have high blood triglyceride levels compared with the control group (rats not administered compound **1**) (Table 4). In experimental type 2 diabetic rats, triglyceride values were found to increase after high-dose ABZ compared with the control group^35^. This information relates to the rise in triglycerides after compound **1** treatment.

**Table 4:** Blood chemistry tests for rats treated with different concentrations of compound 1.

Serum biochemistry tests (mg/dL)	Compound 1 concentration (mg/kg BW)			
	0	175	550	2000
Glucose	218 ± 10.58	219.2 ± 38.69	191.2 ± 33.57	219.6 ± 43.07
Total cholesterol	50.3 ± 4.04	44.2 ± 3.56	45.0 ± 7.14	45.0 ± 3.74
Triglyceride	65.0 ± 27.84	81.2 ± 20.62	95.2 ± 11.69	86.8 ± 7.98
High density lipoprotein	28.67 ± 3.79	26.58 ± 1.62	26.88 ± 4.04	27.4 ± 2.3
Low density lipoprotein	14.0 ± 1.00	11.7 ± 1.51	11.4 ± 1.02	13.2 ± 1.64
Alanine transaminase	31 ± 2.00	33.8 ± 1.64	32.8 ± 5.22	32.4 ± 6.62
Aspartate transaminase	86.3 ± 11.0	103.2 ± 18.4	98.8 ± 14.4	75.6 ± 7.5
Alkaline phosphatase	125 ± 25.12	174 ± 23.00	167 ± 39.66	151 ± 33.50
Creatinine	0.77 ± 0.06	0.72 ± 0.04	0.8 ± 0.1	0.68 ± 0.04
Blood urea nitrogen	18.67 ± 0.58	17.0 ± 1.58	16.6 ± 1.95	18 ± 2.92

Results are reported as mean ± S.E.

**Figure 2:**
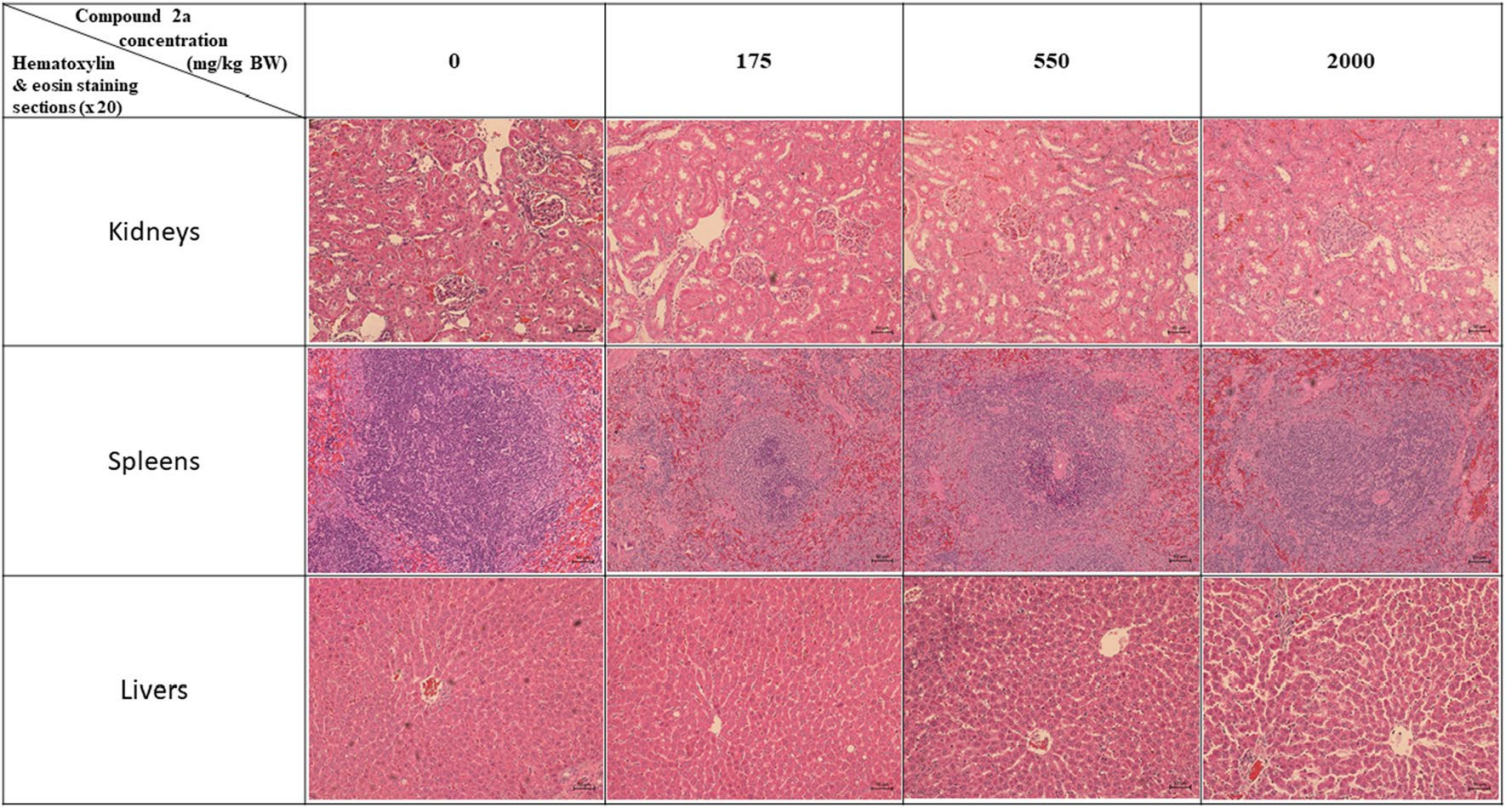
Photomicrographs of kidneys, spleen, and liver sections of rats treated with different concentrations of compound **1.** Total magnification 100x (scale bar for all image is 50 µm).

Liver aspartate transaminase, alanine transaminase, alkaline phosphatase, blood urea nitrogen, and creatinine values were not significantly different among all groups of rats. Hematoxylin and eosin staining of the spleen and kidney of rats that received compound **1** showed similar histopathological shapes and structures to those of the control group. However, there was evidence of hepatocyte dispersion in the liver sections of rats that received 550 and 2000 mg/kg body weight of compound **1**. Some intercellular vacuolization was also observed in liver sections of rats dosed with 2000 mg/kg body weight compound **1** (Fig. 2).

Toxicological screening is very important when exploring the new therapeutic uses for existing molecules^36^. From the acute oral toxicity results, we concluded that compound **1** is a relatively low-acute-toxicity chemical of category 5 with an LD_50_ greater than 2000 mg/kg body weight. This is the highest dose for an acute toxicity test according to the OECD 425 guidelines for the testing of the acute oral toxicity of chemicals, acute toxic class method^37^. Compound **1** showed no effects on the kidney, spleen, or body weight. Compound **1** also caused no mortality in the female rats, which are more sensitive than males, making the results clear. However, the side effects of compound **1** at 550 mg/kg body weight in rats were drowsiness and elevated triglycerides. Previous systematic reviews reported the side effects of anthelmintic drugs on acute liver function^38^. These should be a concern when applying the continued use of the compound in sensitive groups.

### Docking compound **1** with ABZ and IVM targets

**Figure 3:**
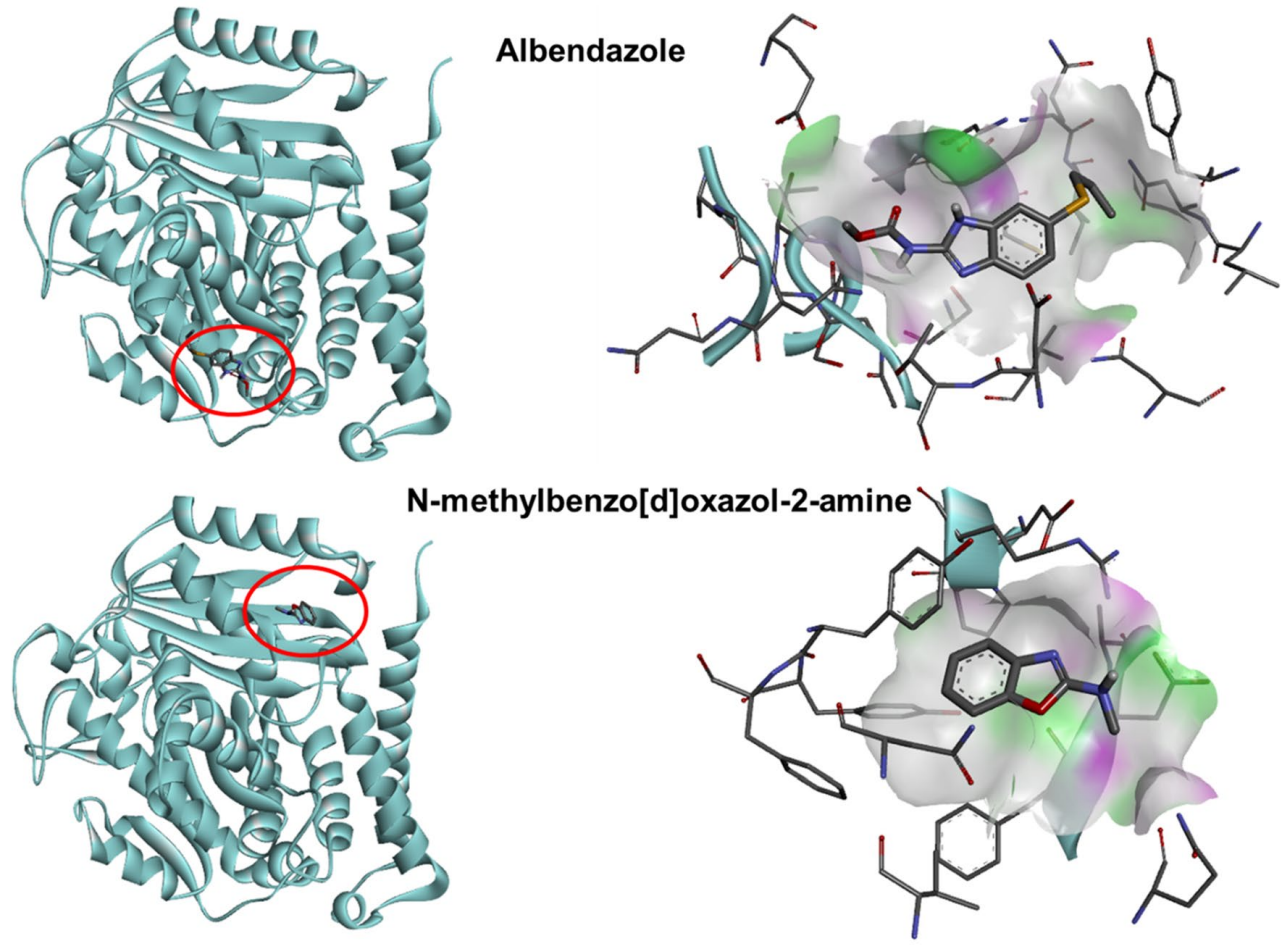
Docking of ABZ and *N*-methylbenzo[d]oxazol-2-amine (compound **1**) to the *T. spiralis* tubulin beta chain. The 3D model of *T. spiralis* tubulin beta chain was generated with SWISS-MODEL using PDB template 6U42. Discovery Studio Visualizer was used to visualize the model. The *T. spiralis* tubulin beta chain is shown as a solid blue ribbon. The top right panel depicts ABZ binding, while the bottom right panel depicts compound **1** binding. Hydrogen bond donors are shown in pink, and hydrogen bond acceptors are shown in green.

To investigate the mechanistic target involved in the anthelmintic activity of compound **1**, molecular modeling and docking methods were used. ABZ’s mode of action is thought to involve *β*-tubulin, as it prevents the production of microtubules^39^. Whereas IVM targets nematode glutamate-gated chloride channels^40^. In our study, the binding energy of compound **1** to both *T. spiralis β*-tubulin and glutamate-gated chloride channels was estimated and compared with those of ABZ and IVM, respectively. Docking ABZ and compound **1** to *T. spiralis* tubulin beta chain resulted in affinity binding energies of − 6.0 kcal/mol and − 5.4 kcal/mol, respectively. Nonetheless, when compound **1** was docked to the same binding cavity as ABZ, the affinity score was − 4.9 kcal/mol. A more negative energy indicates a stronger binding. Thus, the docking results suggested that the binding of compound **1** to tubulin beta chain was weaker than that of ABZ. Both compounds were predicted to fit into distinct binding sites (Fig. 3). However, the EC_50_ of compound **1** was 1.55 µM, which is lower than that of ABZ (3.09 µM), after exposure. This in vitro assay finding demonstrated that compound **1** has an approximately twofold greater activity than ABZ. Therefore, the *T. spiralis* tubulin beta chain might not be the predominant target of compound **1**.

**Figure 4:**
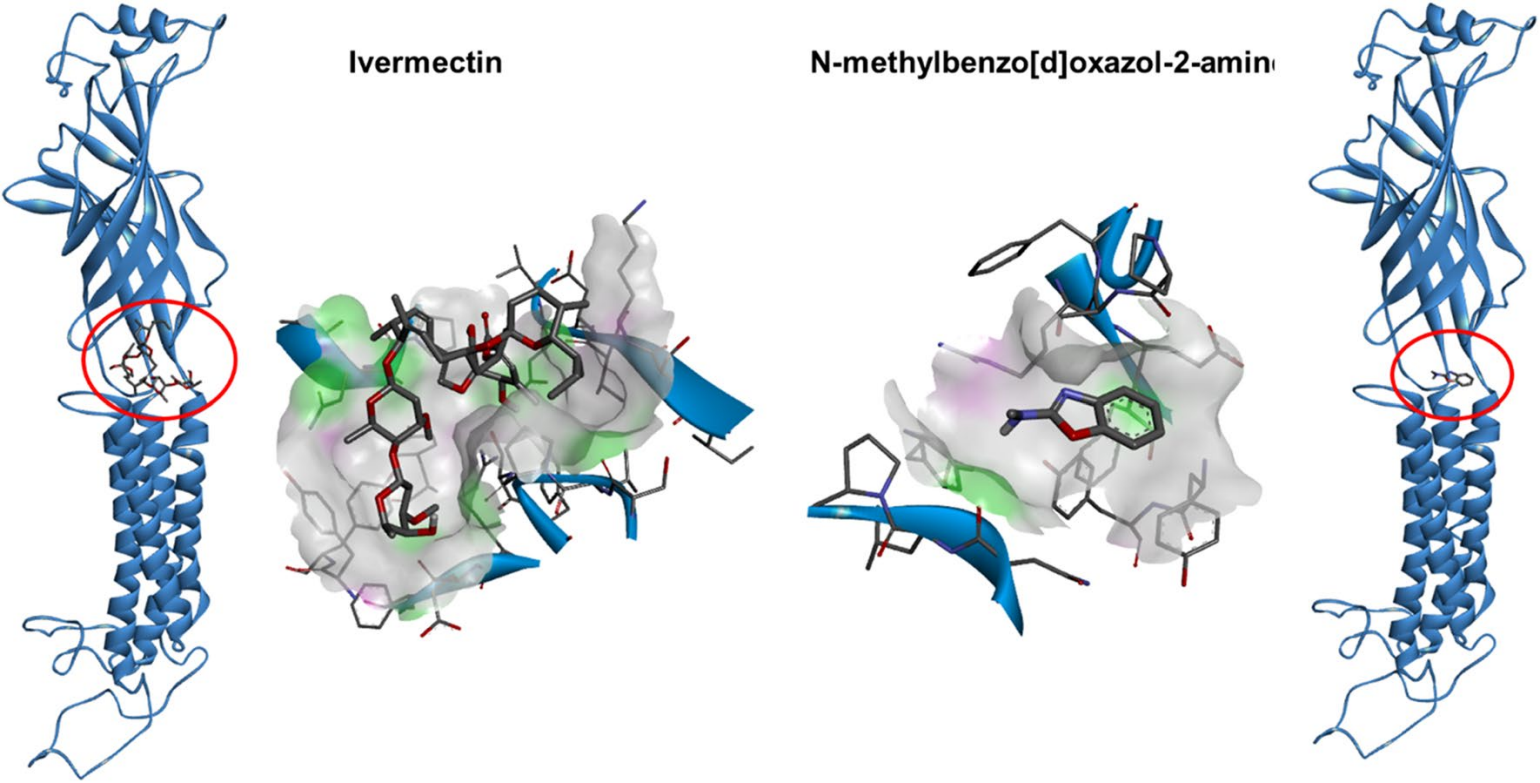
Docking of IVM and *N*-methylbenzo[d]oxazol-2-amine (compound **1**) to *T. spiralis* glutamate-gated channel (GluCl). The 3D model of *T. spiralis* GluCl was generated with SWISS-MODEL using PDB template 4TNW. Discovery Studio Visualizer was used to visualize the model. The *T. spiralis* GluCl is shown as a solid dark blue ribbon. The left panel depicts IVM binding, while the right panel depicts compound **1** binding. Hydrogen bond donors are shown in pink, and hydrogen bond acceptors are shown in green.

*Trichinella spiralis* GluCl docking of IVM and compound **1** resulted in affinity binding energies of − 8.0 kcal/mol and − 4.9 kcal/mol, respectively, suggesting that IVM has a stronger binding affinity than compound **1** (Fig. 4). The greater binding energy of IVM implies that *T. spiralis* GluCl is not the target of compound **1**.

### Metabolomics analysis

**Figure 5:**
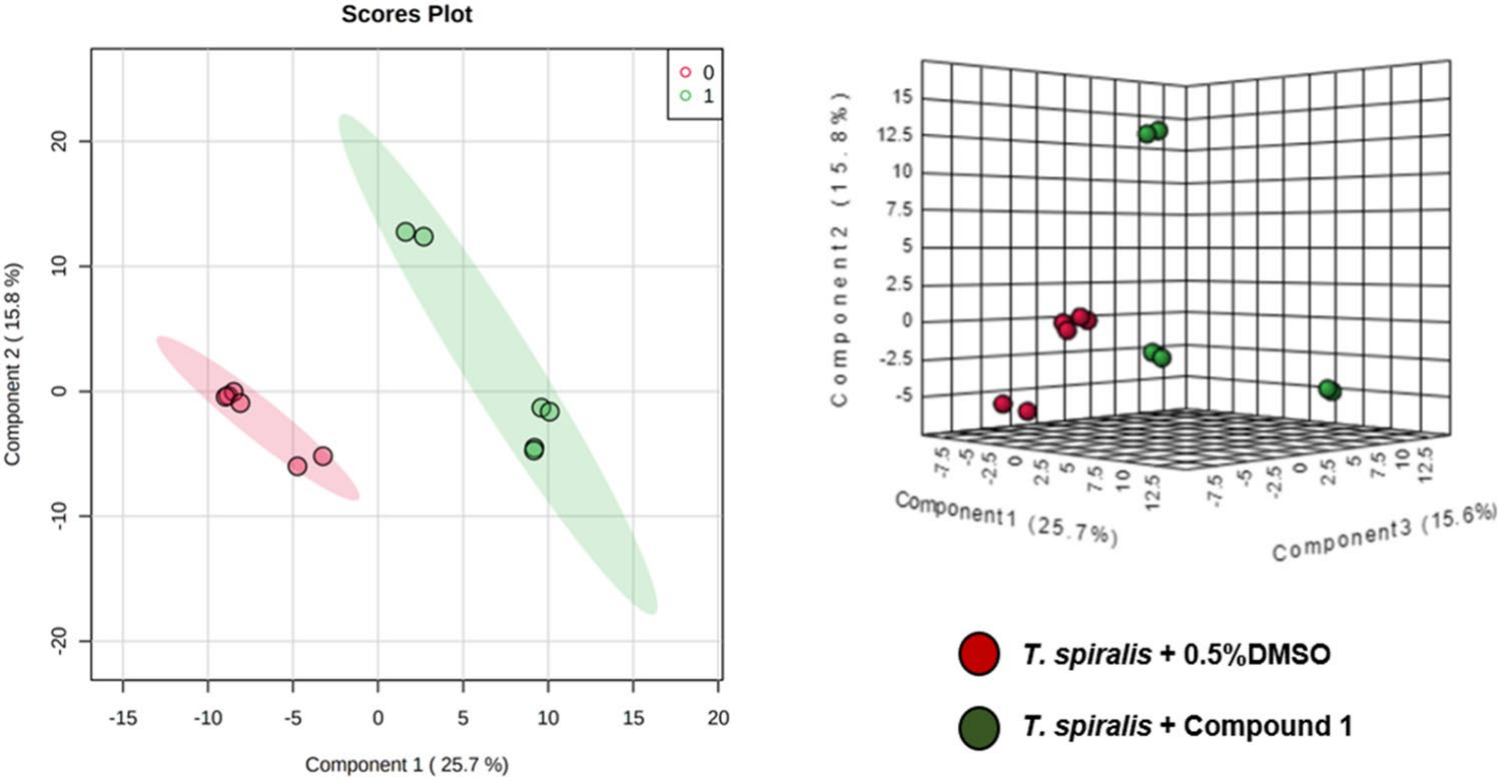
Pairwise analysis of metabolomic data using PLS-DA. Red and green represent data from the control and compound **1** treatments, respectively.

Compound **1** exhibited higher binding energy to the known target proteins of anthelmintic drugs than ABZ and IVM. Metabolomics analysis was therefore used to explain the effects of compound **1** on *T. spiralis* adult worms. The metabolite profiles of 0.5% DMSO (control) and compound **1** EC_50_-treated *T. spiralis* adult worms were studied. Partial least squares discriminant analysis (PLS-DA) was employed for data analysis (Fig. 5).

**Figure 6:**
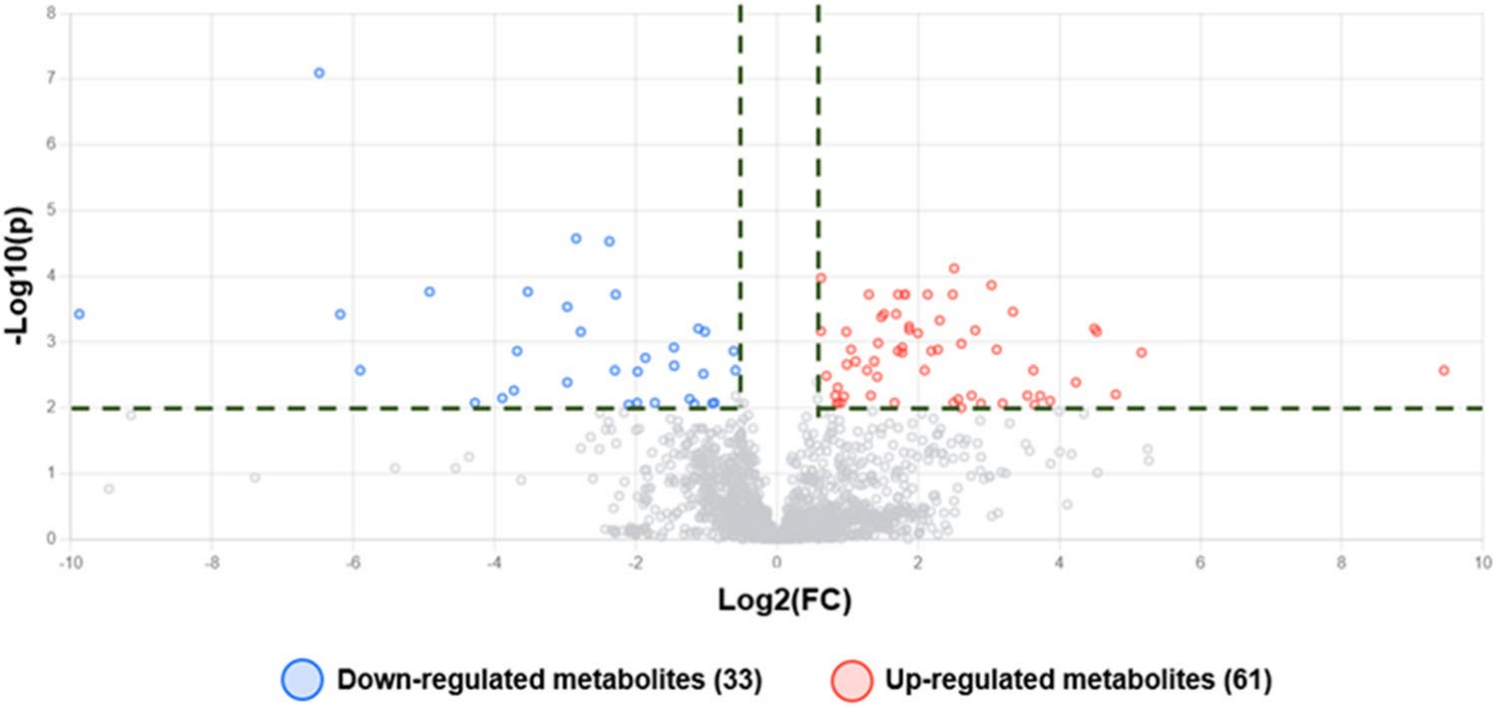
Volcano plots showing differential metabolites of *T. spiralis* adult worms after compound **1** treatment. Horizontal lines represent p-values equal to 0.01. Vertical lines represent fold changes equal to 1.5 and − 1.5. Blue and red dots refer to down- and up-regulated metabolites, respectively.

The results revealed that the metabolite profile of *T. spiralis* adult worms after compound **1** exposure was clearly different those of the control. A total of 13,469 features were observed by mass spectrometric analysis. There were 94 differential features of *T. spiralis* adult worms after compound **1** exposure. Among them, 9 metabolites were identified by METLIN database. The volcano plot in Fig. 6. was used to show statistical significance (p-value) versus magnitude of change (fold change). Using the criteria of p-value < 0.01 and fold change ≥ 1.5, we identified 94 differential features after compound **1** exposure. Among them, 61 and 33 were up- and down-regulated, respectively. Metabolite identification using the METLIN database revealed 3 and 6 up- and down-regulated metabolites, respectively (Tables 5, 6, and S1).

**Table 5:** Up-regulated metabolites of *T. spiralis* after compound 1 exposure.

No..	Potential metabolites	*m/z*	Retention time (min)	Mass error (ppm)	Adduct	Mode	FC	P-value
1	4alpha-hydroxymethyl-5alpha-cholesta-8-en-3beta-ol	381.3527	15.56	0	M+H-2H_2_O	Positive	35.91	0.0015
2	Isoleucyl-methionine	307.1063	0.84	0	M+2Na-H	Positive	2.52	0.0065
3	MG(18:0/0:0/0:0)	341.3055	13.52	0	M+H-H_2_O	Positive	1.54	0.0001

**Table 6:** Down-regulated metabolites of *T. spiralis* after compound 1 exposure.

No.	Potential metabolites	*m/z*	Retention time (min)	Mass error (ppm)	Adduct	Mode	FC	P-value
1	2-amino-14,16-dimethyloctadecan-3-ol	314.3420	14.93	1	M+H	Positive	59.81	0.0026751
2	Phytosphingosine	318.3002	1.33	0	M+H	Positive	3.94	0.0083208
3	Palmitamide	256.2632	1.33	0	M+H	Positive	3.93	0.0027868
4	PC(18:0/22:4(7Z,10Z,13Z,16Z))	838.6310	15.28	1	M+H	Positive	3.64	0.0017547
5	Benzoyl glucuronide (benzoic acid)	281.0660	0.91	0	M+H-H_2_O	Positive	2.74	0.0022911
6	PE(16:0/22:6(4Z,7Z,10Z,13Z,16Z,19Z))	763.5167	19.74	2	M+	Positive	1.85	0.0083208

According to the fold-change values, 4alpha-hydroxymethyl-5alpha-cholesta-8-en-3beta-ol was the most up-regulated metabolite after compound **1** exposure. It has been found as an ergosterol intermediate in sterol biosynthetic pathway of *Leishmania* spp. Amphotericin B is the compound of choice for leishmaniasis treatment. One of its mechanisms is rapidly alteration of lipid metabolism of the *Leishmania* parasite^41^. In addition, several antiparasitic drugs interfere the sterol biosynthetic pathway such as zaragozic acids, quinuclidines and bisphosphonates for treating human African trypanosomiasis, Chagas disease, and leishmaniasis^42,43^. The up-regulation of 4alpha-hydroxymethyl-5alpha-cholesta-8-en-3beta-ol after compound 1 exposure might interfere sterol biosynthetic pathway and demise of *T. spiralis*. MG(18:0/0:0/0:0) was also up-regulated after compound 1 exposure. This compound is monoacylglyceride, an ester of the trihydric alcohol glycerol and a long-chain fatty acid. Monoacylglycerol lipase is an enzyme producing monoacylglycerides. This enzyme is a target protein of an antiplasmodial compound, salinipostin A. The alteration of monoacylglyceride level could affect vitality of *P. falciparum*^44^. In addition, a monoacylglyceride from Nordic seaweeds demonstrated as a major anti-parasitic compound against *Ascaris suum*^45^. Therefore, altered monoacylglyceride level may have an effect on *T. spiralis* survivability similar to *P. falciparum*.

**Figure 7:**
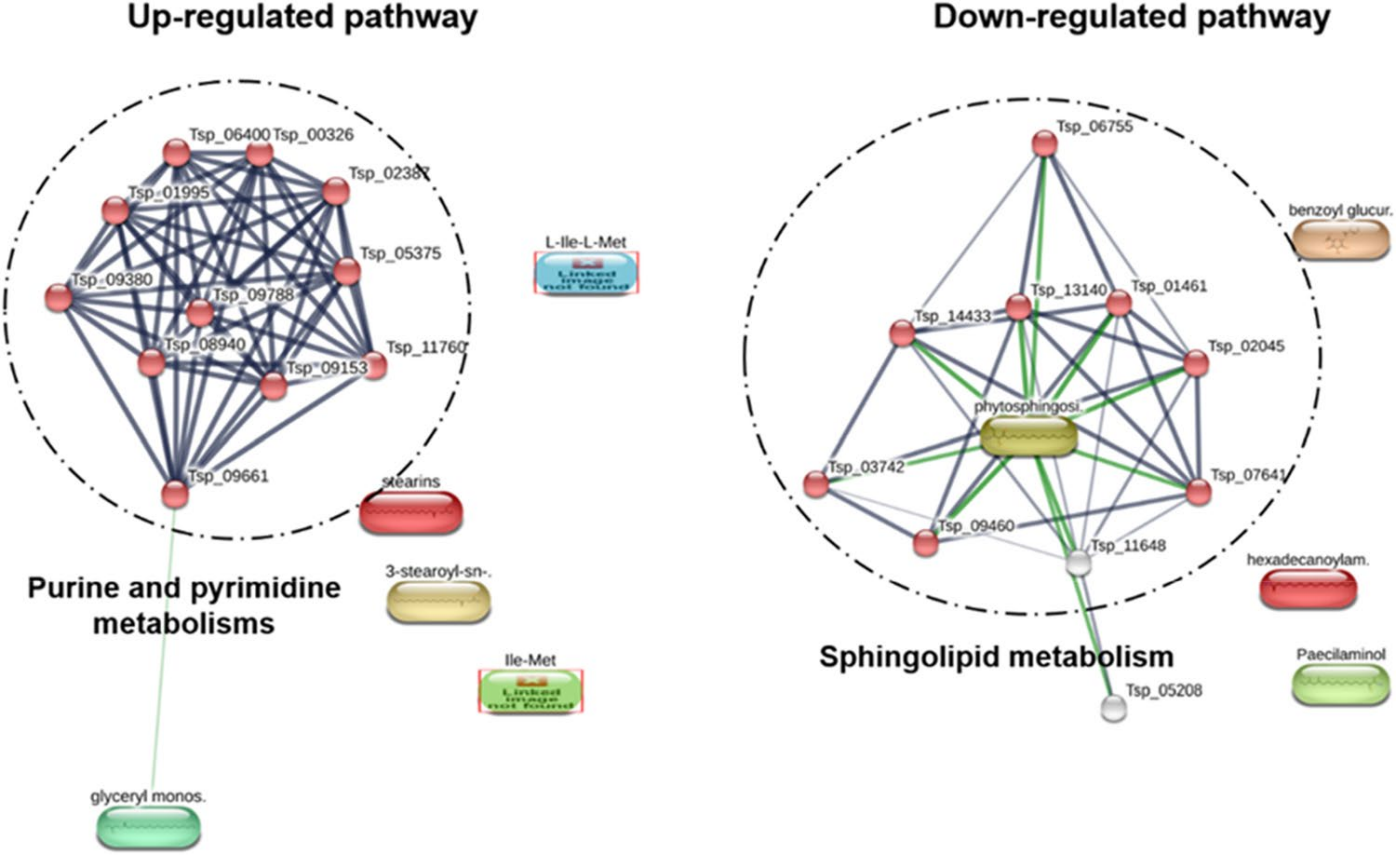
Pathway analysis of significantly altered metabolites after compound **1** exposure. Circles indicate the first shell of interactors related to the predicted pathway. Purine and pyrimidine metabolisms, and sphingolipid metabolism were predicted to be the related pathway with the false discovery rate of 2.27e−21 and 1.26e−19, respectively.

The 2-amino-14,16-dimethyloctadecan-3-ol and phytosphingosine were down-regulated metabolites after compound **1** exposure involving in sphingolipid biosynthesis. The 2-amino-14,16-dimethyloctadecan-3-ol is natural analogs of sphinganine and 1-deoxysphinganine which could control sphingolipid production^46^. While phytosphingosine is a sphingoid base, a fundamental structure for generating sphingolipids. Phytosphingosine is abundant in plants and fungi and present in animals^47^. No information relating to 2-amino-14,16-dimethyloctadecan-3-ol and phytosphingosine functions in the parasite is available. However, the alteration of sphingolipid level could inhibit *P. falciparum* development^48^. The development of *T. spiralis* may be inhibited by interfering sphingolipid biosynthesis after exposure to compound **1**. Palmitamide was also down-regulated after compound **1** exposure, and this compound has inflammatory properties^49^. In general, helminths are successful at modulating their host’s immune response^50^. *Schistosoma mansoni* secretes an anti-inflammatory molecule to modulate and circumvent the host's innate and adaptive immune responses^51^. The reduction in palmitamide may affect the parasite’s ability to modulate its host’s immunity, potentially restoring the host’s defense mechanism^52–54^. The differential metabolites were subjected to pathway analysis using the STITCH bioinformatics tool (Fig. 7). The significantly up-regulated metabolic pathway was purine and pyrimidine metabolisms, while the most down-regulated was sphingolipid metabolism. The purine and pyrimidine metabolisms are important pathways generating purine and pyrimidine molecules for DNA replication, RNA synthesis, and cellular bioenergetics. The imbalance in purine and pyrimidine metabolisms are linked with inability to maintaining cell homeostasis^55^. Accordingly, the elevation of these mechanisms promotes uncontrolled growth of tumors and is one of characteristic of cancer^56^. Since the crucial role of the purine and pyrimidine metabolic, they have been proposed as antiparasitic drug targets in *Plasmodium falciparum*^57^ and *Schistosoma* spp^58^. The highly regulated purine and pyrimidine metabolisms in *T. spiralis* after compound **1** exposure may bring imbalance of nucleotide molecule, failure to maintain parasite cell homeostasis and cause death of the parasite. For down-regulation of sphingolipid metabolism, the alteration of sphingolipid level could affect parasite survival as described above.

**Figure 8:**
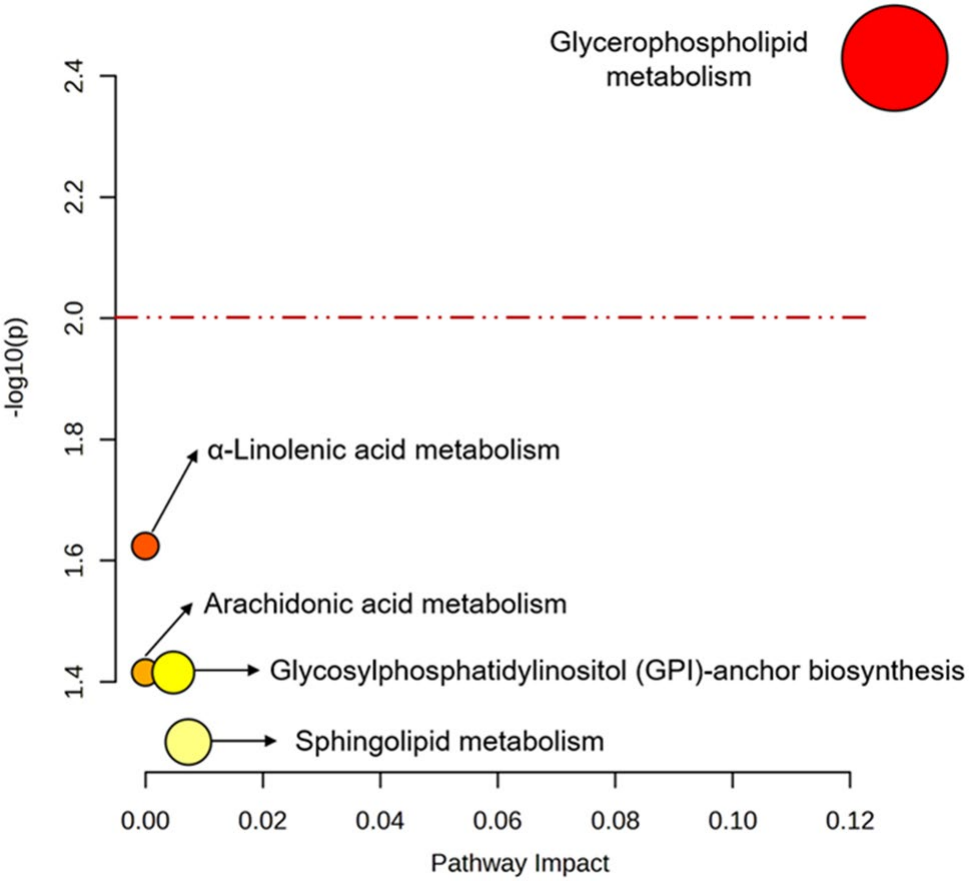
Metabolite annotation and pathway analysis using MetaboAnalyst. Red line represents the statistically significant at p-value < 0.01.

Further pathway analysis was performed on all altered metabolites using MetaboAnalyst (Fig. 8), and the most significantly altered pathway for all metabolites was glycerophospholipid metabolism. Both bioinformatics tools confirmed that glycerophospholipid metabolism plays an important role in compound **1** activity mechanisms. Glycerophospholipids, which are fatty acid diglycerides with a phosphatidyl ester molecule linked to the terminal carbon, are components of cellular or vesicle membranes^59^. The up-regulated glycerophospholipid metabolic pathway was shown to play an important role in the low-oxygen supply stress response in *Saccharomyces cerevisiae*^60^. In addition, glycerophospholipid metabolism was reported to be a potential pathway for antiparasitic drug development for *Trichinella papuae*^61^, *P. falciparum*^62^, *Leishmania*, *Trypanosoma brucei,* and *Trypanosoma cruzi*^63^. Therefore, *T. spiralis* glycerophospholipid metabolism might be affected by the stress generated by compound **1**. The down-regulation of sphingolipid metabolism was also observed following compound **1** treatment. Sphingolipids are a class of lipids containing a sphingoid base backbone and the organic aliphatic amino alcohol sphingosine. Sphingolipids are abundant membrane components of pathogenic protozoans such as *Leishmania major*^64^, *Trypanosoma brucei*^65^, *P. falciparum*^66^, and *Giardia lamblia*^67^. Sphingolipid metabolism potentially contributes to parasite survival and host defense^68^. Moreover, sphingolipid production is necessary for cell cycle progression and cell survival in *T. brucei*, but it is not necessary for the regular movement of proteins associated with the flagellar membrane or the creation of lipid rafts^69^. Therefore, *T. spiralis* parasite survival may be impacted by the down-regulation of the sphingolipid pathway.

## Conclusion

We investigated the anthelmintic activity and toxicity of compound **1** in this study using rodents and *T. spiralis*. Through this study, we found that compound **1** has good in vitro anthelmintic activity and acceptable toxicity in an animal model. However, it demonstrated lower in vivo anthelmintic efficacy compared with ABZ. The metabolomics results showed that purine and pyrimidine metabolisms and sphingolipid metabolism were significantly affected by compound **1**. Further studies into its absorption, bioavailability, pharmacokinetics, and efficacy would help to understand this lack of in vitro and in vivo correlation. The information could also be advantages for further development of compound **1** as an anthelmintic**.**

### Supplementary Information

Supplementary Table S1.

Supplementary Figure S1.

## Data Availability

The datasets generated and/or analysed during the current study are available in the Science Data Bank repository (https://www.scidb.cn/anonymous/Qk5CYnFh).
