# Supplementary figures and images for: Anthelmintic efficacy evaluation and mechanism of N-methylbenzo[d]oxazol-2-amine
Source: Sci Rep. 2023 Dec 21;13:22840. doi: 10.1038/s41598-023-50305-y (PMC10739888; doi:10.1038/s41598-023-50305-y)

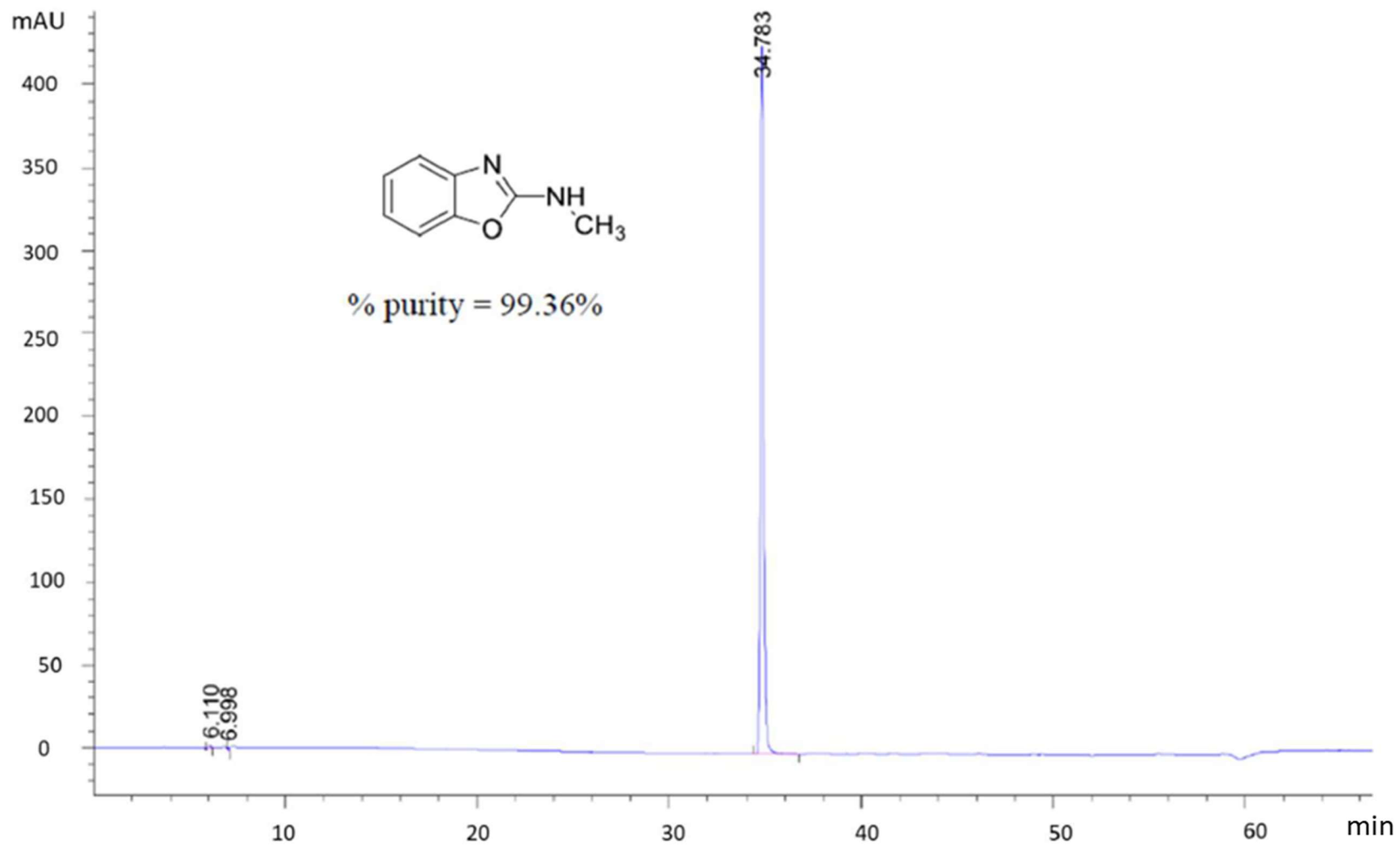

**Fig. S1** The HPLC trace of compound 1 using 254nm absorbance. The purity was 99.36%.

Supplement: Supplementary file 2 — Supplementary Figure S1. [file 41598_2023_50305_MOESM2_ESM.pdf]
